# The Virulence Regulator Rns Activates the Expression of CS14 Pili

**DOI:** 10.3390/genes7120120

**Published:** 2016-12-08

**Authors:** Maria Del Rocio Bodero, George Patrick Munson

**Affiliations:** Department of Microbiology and Immunology, University of Miami Miller School of Medicine, Miami, FL 33136, USA; mclouse413@gmail.com

**Keywords:** enterotoxigenic *E. coli*, ETEC, pili, Rns, activator

## Abstract

Although many viral and bacterial pathogens cause diarrhea, enterotoxigenic *E. coli* (ETEC) is one of the most frequently encountered in impoverished regions where it is estimated to kill between 300,000 and 700,000 children and infants annually. Critical ETEC virulence factors include pili which mediate the attachment of the pathogen to receptors in the intestinal lumen. In this study we show that the ETEC virulence regulator Rns positively regulates the expression of CS14 pili. Three Rns binding sites were identified upstream of the CS14 pilus promoter centered at −34.5, −80.5, and −155.5 relative to the Rns-dependent transcription start site. Mutagenesis of the promoter proximal site significantly decreased expression from the CS14 promoter. In contrast, the contribution of Rns bound at the promoter distal site was negligible and largely masked by occupancy of the promoter proximal site. Unexpectedly, Rns bound at the site centered at −80.5 had a slight but statistically significant inhibitory effect upon the pilin promoter. Nevertheless, this weak inhibitory effect was not sufficient to overcome the substantial promoter activation from Rns bound to the promoter proximal site. Thus, CS14 pili belong to a group of pili that depend upon Rns for their expression.

## 1. Introduction

First identified nearly 50 years ago as the causative agent of non-vibrio cholera, enterotoxigenic *Escherichia coli* (ETEC) remains a leading cause of diarrheal disease [1,2,3,4,5]. Approximately 280 million people are sickened by ETEC annually and in low-income nations the enteric pathogen is estimated to kill between 300,000 and 700,000 infants and children [6,7,8]. Travelers to high-risk areas may suffer infection rates as high as 50% [9]. High infection rates have also been reported for U.S. military personnel stationed within or transiting through risk areas [10]. Sporadic ETEC outbreaks also occur in developed nations, including the United States [11,12]. Although several are under development, currently there are no Food and Drug Administration (FDA)-approved ETEC vaccines [13,14]. 

Two types of enterotoxins have been extensively characterized, either of which is capable of causing diarrhea [15]. One is the heat-labile toxin which is a multimeric toxin composed of one A subunit and five B subunits. The B subunits mediate binding to host cell receptors to facilitate delivery of the toxin to the cytosol of host cells [16,17]. Once inside a host cell, the A subunit ADP ribosylates Gsα which results in the deregulation of adenylate cyclase and the overproduction of cAMP [18]. The other type of enterotoxin is the heat-stable toxin, which is a small peptide that mimics the hormone guanylin to activate guanylate cyclase C. This causes the overproduction of cGMP [19]. In both cases the disruption of cyclic nucleotide homeostasis alters the transport of ions across the apical membranes of epithelial cells, leading to profuse watery diarrhea [15]. 

ETEC virulence is also dependent upon afimbrial adhesins and pili that mediate attachment to host cell receptors in the lumen of the intestine [20,21,22,23]. To date, 24 different types of adhesive pili have been characterized [7,22,24]. Although there is significant heterogeneity among ETEC strains with regards to pili, Rns (CfaD, CfaR) is known or thought to activate the expression of nearly half of all known ETEC pili [22,25,26,27,28,29,30]. Rns is a 31 kDa protein and a member of the AraC/XylS family of transcriptional regulators [31,32]. Like most family members, the carboxy-terminal domain of Rns contains two helix-turn-helix motifs and each is thought to engage the major groove of the DNA helix [28]. Unlike homodimeric transcription factors whose symmetry is mirrored in palindromic DNA binding sites, each helix-turn-helix motif of Rns is unique. Thus, Rns binding sites are asymmetrical, as is the case of the well-characterized CS1 pilus promoter *cooBp* [28]. 

DNase I footprinting revealed two Rns binding sites upstream of the Rns-dependent transcription start site of *cooBp*, one centered at −40.5 and the other −110.5. Mutagenesis of either site decreased Rns-dependent expression from *cooBp*; however, mutagenesis of the promoter proximal site produced a larger defect than mutagenesis of the promoter distal site [28]. A similar arrangement of binding sites was identified at the CS17, CS19, and PCF071 pilus promoters [27]. As with the CS1 promoter, occupancy of the promoter proximal sites contributed more towards transcriptional activation of the CS17, CS19, and PCF071 promoters than occupancy of the promoter distal sites. We also note that Rns has also been shown to regulate the expression of genes that do not encode pilins. However, the contribution of these other genes towards ETEC pathogenesis remains unclear [33,34,35].

Although Rns binding sites were also identified upstream of the CFA/I pilus promoter, to date CS1, CS17, CS19, and PCF071 remain the most extensively characterized Rns-activated pilus promoters [27,28,34]. In terms of phylogeny, CS1, CS17, CS19, PCF071, and CFA/I pili belong to the class 5 group—more recently termed the α-clade—of adhesive pili [22,36]. CS14 is a less-well-characterized member of this group, with little known about its expression. In this study we expand the known repertoire of Rns-regulated genes by showing that Rns positively regulates the expression of CS14 pili and we identify three Rns binding sites upstream of the CS14 pilus promoter. Site-directed mutagenesis of each binding site indicates that Rns bound at the promoter proximal site is responsible for the majority of Rns-dependent transcription from the pilin promoter and largely masks the contributions of the two other binding sites.

## 2. Materials and Methods

### 2.1. Strains and Plasmids

WS3294A, provided by S. Savarino (Naval Medical Research Center, Silver Spring, MD, USA), is a clinical isolate of ETEC that infects humans [36]. Bacterial strains are listed in Table 1. CS14 promoter fragments were amplified from WS3294A with primer pairs 542/39, 542/554, 771/554, and 772/554. Oligonucleotide primers are listed in Table 2. Enzymes used in this study were purchased from New England Biolabs, Ipswich, MA, USA. The polymerase chain reaction (PCR) products were then digested with the restriction enzymes (RE) BamHI and EcoRI then ligated into the same sites of pHKLac1 to construct pCS14Lac1, pCS14Lac2, pCS14Lac6, and pCS14Lac5, respectively. Plasmid pHKLac1 is a promoterless Lac reporter and integration plasmid with a R6Kγ *pir*-dependent origin of replication that has previously been described [35]. It carries *attP*_HK022_ for Int_HK022_-mediated integration at *attB*_HK022_ in *pir* deficient hosts and *aadA* for selection with spectinomycin and streptomycin [37]. Rns binding sites *csuB1o*, *csuB2o*, and *csuB3o* were mutagenized by inverse PCR of intermediate cloning plasmids with primers 566/567, 564/565, and 769/770 to generate restriction sites for SpeI, KpnI, and AgeI, respectively. The mutagenized promoter fragments were then cloned into pHKLac1 as per above. All promoter fragments were sequenced to verify the absence of PCR unintended mutations. Each reporter plasmid was then integrated into the chromosome of MC4100 and single integrants were verified by colony PCR as previously described [37]. Plasmids pGPMRns and pGPMRns<Tn>2 have been previously described [35,38].

### 2.2. β-Galactosidase Assays

To determine the level of Rns-dependent transcription from the CS14 promoter, Lac reporter strains were transformed with pGPMRns (*rns*^+^). Reporter strains were transformed with pGPMRns<Tn>2 (*rns::kan*) a plasmid that carries the *rns* gene disrupted by a kanamycin (kan) resistance cassette to determine the level of Rns-independent transcription. Transformed reporter strains were cultured aerobically at 37 °C in Lysogeny broth (LB) medium with 100 µg/mL ampicillin and harvested in late log phase. Cells were lysed and assayed for β-galactosidase activity as previously described [28]. 

### 2.3. Purification of MBP-Rns

MBP-Rns was purified as previously described [27,28]. In brief, strain KS1000/pRare2/pMBPRns1 was cultured aerobically at 37 °C in LB medium containing 0.2% wt/vol glucose, 30 μg/mL chloramphenicol, and 100 μg/mL ampicillin. KS100 was obtained from New England Biolabs, Ipswich, MA, USA. To induce the expression of MBP-Rns, isopropyl β-D-1-thiogalactopyranoside was added to a final concentration of 300 µM during mid-log phase and the bacteria were cultured for an additional 3–4 h at 30 °C. Bacteria were collected by centrifugation and concentrated ≥100-fold in lysis buffer (10 mM TrisCl, pH 7.6, 200 mM NaCl, 1 mM EDTA, 0.5 mM CaCl_2_, 10 mM β-mercaptoethanol, 100 µg/mL DNase I) at 4 °C. Bacteria were lysed in a French press (Thermo Electron Corporation, Needham Heights, MA, USA) MBP-Rns was collected from the soluble fraction by amylose affinity column chromatography and eluted with elution buffer (10 mM TrisCl, pH 7.6, 200 mM NaCl, 1 mM EDTA, 15% vol/vol glycerol, 10 mM β-mercaptoethanol, 10 mM maltose). The eluent was dialyzed against 10 mM TrisCl, pH 7.6, 50 mM KCl, 1 mM β-mercaptoethanol, 30% vol/vol glycerol and stored at −80 °C. Protein concentration was determined by the Bradford method relative to a standard curve of bovine serum albumin.

### 2.4. DNaseI Footprinting

CS14 promoter fragments were labeled with ^32^P by PCR with one primer labeled at its 5’ terminus and one unlabeled primer. Labeled PCR products were purified by recovery from non-denaturing acrylamide gels and equilibrated with MBP-Rns in 10 mM TrisCl, pH 7.6, 50 mM KCl, 1 mM DTT, 0.4 mM MgCl_2_, 0.2 mM CaCl_2_, 2 ng/µL polydI-dC, 10 µg/mL bovine serum albumin [27]. The solution was equilibrated for 10–20 min at 37 °C then DNase I was added to a final concentration of 100 ng/µL for 1 min at 37 °C. Cleavage was terminated by addition of 10 volumes of 570 mM NH_4_OAc, 50 µg/mL tRNA, 80% vol/vol ethanol. Precipitated DNA was washed with 70% vol/vol cold ethanol, dried, then suspended in 4 µL 80% vol/vol formamide, 50 mM Tris-Borate, pH 8.3, 1 mM EDTA, 0.1% wt/vol xylene cyanol and bromophenol blue. Denatured DNA fragments were separated on sequencing gels and imaged by exposure to phosphorimager plates (GE Healthcare, Chicago, IL, USA). Sequence ladders were generated by the Maxam-Gilbert method [39]. 

### 2.5. Determination of Transcription Start Sites

Rns-dependent and -independent transcription start sites were mapped as previously described [27]. In brief, total RNA was isolated from reporter strain GPM1124 transformed with pGPMRns or pGPMRns<Tn>2 by phenol extraction [35]. Subsequently, ca. 2.4 picomoles of ^32^P end-labeled oligonucleotide 346 was combined with 63–65 μg of total RNA and 0.8 mM deoxynucleotides. The oligonucleotide was extended with SuperScript™ III Reverse Transcriptase according to the supplier’s protocol (Invitrogen, Carlsbad, CA, USA). Primer extension products were separated on DNA sequencing gels alongside Maxam-Gilbert sequencing ladders and imaged by exposure to phosphorimager plates.

## 3. Results

### 3.1. Rns Activates the CS14 Pilus Promoter

In terms of phylogeny, CS14 pili belong to the class 5 group—more recently termed the α-clade—of adhesive pili [22,36]. The expression of some—but not all—members of this clade is dependent upon Rns [25,27,28,40]. Therefore, we sought to determine if CS14 pili belong to the subgroup of class 5 pili whose expression is Rns-dependent or the subgroup whose expression is Rns-independent. To accomplish this, we cloned a DNA region spanning −249 to +440, relative to the transcription start site of *csuB*, from ETEC strain WS3294A into a promoterless Lac reporter to construct pCS14Lac2. The reporter plasmid was then integrated into the *attB*_HK022_ site of Lac^−^ K-12 strain MC4100 to construct reporter strain GPM1132. This strain produced only 7 ± 0.5 (*n* = 3) Miller units of β-galactosidase when transformed with pGPMRns<Tn>2 (*rns::kan*). However, 2050 ± 207 (*n* = 3) Miller units were produced when the GPM1132 was transformed with the Rns expression plasmid pGPMRns. The 292-fold increase in β-galactosidase expression from the CS14 promoter *csuBp* was statistically significant (*p* < 0.001, Student’s unpaired *t*-test). Thus, these results clearly demonstrate that Rns activates the CS14 promoter and places CS14 pili into the Rns-dependent subgroup of class 5 pili. 

### 3.2. Determination of the csuBp Transcription Start Site

**Figure 1:**
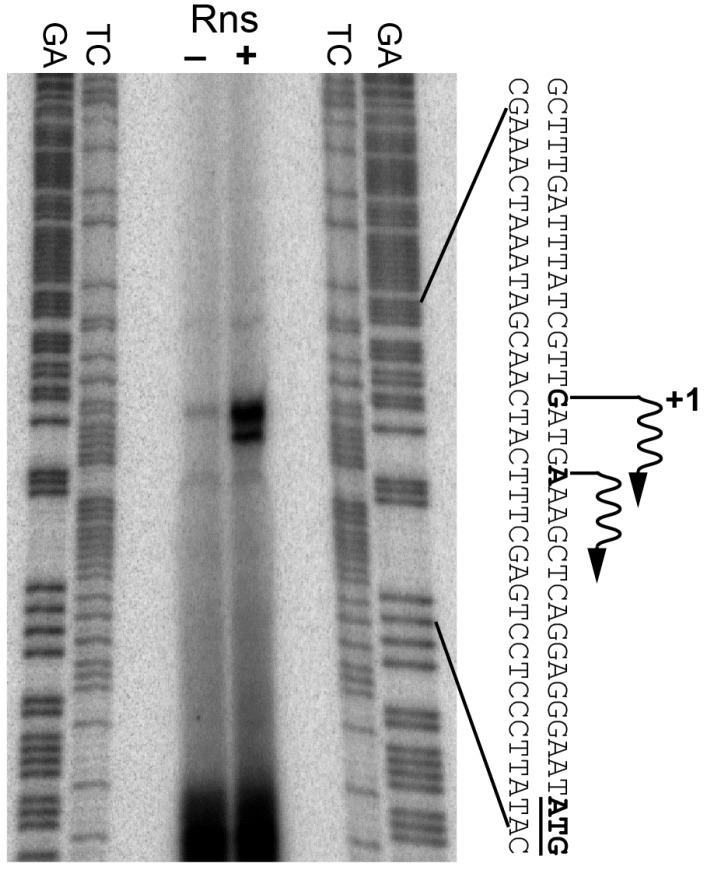
Identification of the Rns-dependent transcription start site. The transcription start site of the CS14 pilus promoter was mapped by primer extension of mRNA isolated from *rns*^+^ and *rns::kan* strains. Transcription start sites and the start codons of *csuB* are shown in bold. Wavy arrows indicate the direction of transcription. Lanes labeled GA and TC contain Maxam-Gilbert sequencing ladders.

Since our β-galactosidase assays indicated that Rns activates *csuBp*, we next used primer extension to map the promoter’s Rns-dependent transcription start site. Our analysis revealed one primer extension product that mapped to a deoxyguanosine 21 bp upstream of the start codon for *csuB* (Figure 1). A second primer extension product mapped to a deoxyadenosine 17 bp upstream of the start codon. Digital densitometry revealed that the product mapping to the deoxyguanosine was approximately four times more abundant than the one mapping to the deoxyadenosine. Therefore, we consider the deoxyguanosine at −21 to be the primary Rns-dependent transcription start site of *csuBp*. In the absence of Rns, the primary transcription start site was barely detectable. The substantially reduced abundance of the primer extension product is consistent with the low level of β-galactosidase expression observed with the *rns::kan* Lac reporter strain reported above. 

### 3.3. Identification of Rns Binding Sites by DNase I Footprinting

**Figure 2:**
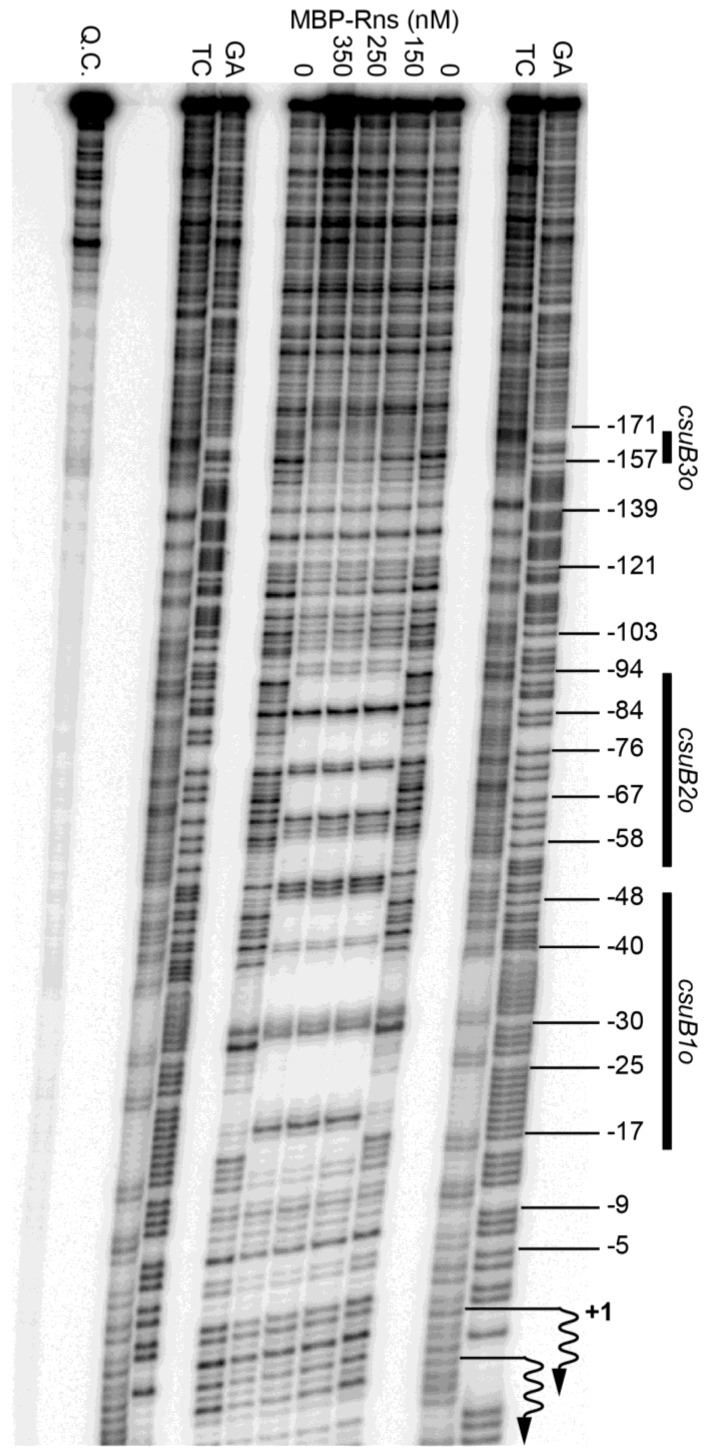
Identification of Rns binding sites. DNase I footprints of MBP-Rns bound the noncoding strand of the CS14 promoter labeled with ^32^P at its 5′ end. Numbering is relative to the Rns-dependent transcription start site, denoted by wavy arrows. Lanes labeled TC and GA contain Maxam-Gilbert sequencing ladders. Q.C. denotes a template quality control that was not digested with DNase I.

We next used DNase I footprinting to determine if Rns binds at or near the CS14 pilus promoter. As with previous studies, we used a purified fusion protein consisting of MBP at the amino terminus of Rns because the native form of Rns, like several other AraC/XylS family members, is too insoluble for this type of in vitro assay [27,28,34,35,41]. The solubility of Rns is significantly enhanced by the addition of MBP, which also facilitates its purification by affinity chromatography [28]. Importantly, the fusion protein has been shown to be functional both in vivo and in vitro [28]. Thus, the addition of MBP to the amino-terminus of Rns does not alter its specific interactions with DNA. When bound to a DNA fragment carrying the CS14 promoter, the fusion protein produced a DNase I footprint that extended from approximately −17 to −91 relative to the primary transcription start site of *csuBp* (Figure 2). This extended footprint is roughly twice as large as the footprint of the fusion bound to a single site [28,34,35]. The DNase I hypersensitive sites at −52 and −53 likely delineate the boundary between sites *csuB1o* and *csuB2o* (Figure 2). 

An additional region of protection was observed from ca. −145 to −171 (Figure 2). However, protection in this upstream region was not apparent with 150 nM MBP-Rns and did not appear to reach saturation even with 350 nM MBP-Rns. In contrast, the two binding sites between −17 and −91 were fully saturated with 150 nM MBP-Rns because the extended footprint was unchanged by higher concentrations of protein. This suggests that the distal upstream binding site *csuB3o* may be a low-affinity binding site relative to the two more promoter-proximal sites. 

### 3.4. Analyses of Rns Binding Sites

**Figure 3:**
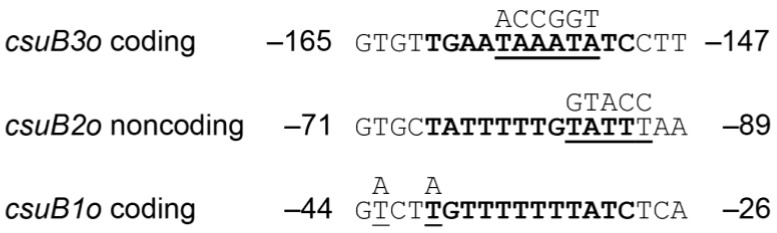
Sequence alignment of Rns binding sites. Within each DNase I footprint, sequences similar to the 12 bp Rns binding site motif were identified and are shown in bold. Nucleotides selected for site-directed mutagenesis are underlined and the mutations are shown above each sequence. Numbering is relative to the Rns-dependent transcription start site.

Sequence analysis of each binding site revealed that each contains 12 bp sequences with a similarity to previously reported Rns binding sites (Figure 3) [27,28,34,35,41]. These consensus-like sequences are centered at −34.5, −80.5, and −155.5 with respect to the Rns-dependent transcription start site of *csuBp*. Because Rns binding sites are not palindromic, Rns binding site motifs may appear on either DNA strand [28]. For sites *csuB1o* and *csuB3o*, the conserved motifs are found on the coding strand (Figure 3). In contrast, the motif is present on the noncoding strand within site *csuB2o*. To analyze the function of these sites in vivo, each was subjected to oligonucleotide-directed mutagenesis to alter one or more nucleotides within the 12 bp motifs (Figure 3). The number and position of each mutation was determined by the sequence of selected endonuclease restriction sites (SpeI within *csuB1o*, KpnI within *csuB2o*, and AgeI within *csuB3o*), the generation of which facilitated screening for mutagenized binding sites. The resulting constructs were cloned into Lac reporter plasmids which were subsequently integrated into the *attB*_HK022_ site of MC4100 so that the function of each binding site could be evaluated in vivo (Table 1). 

**Figure 4:**
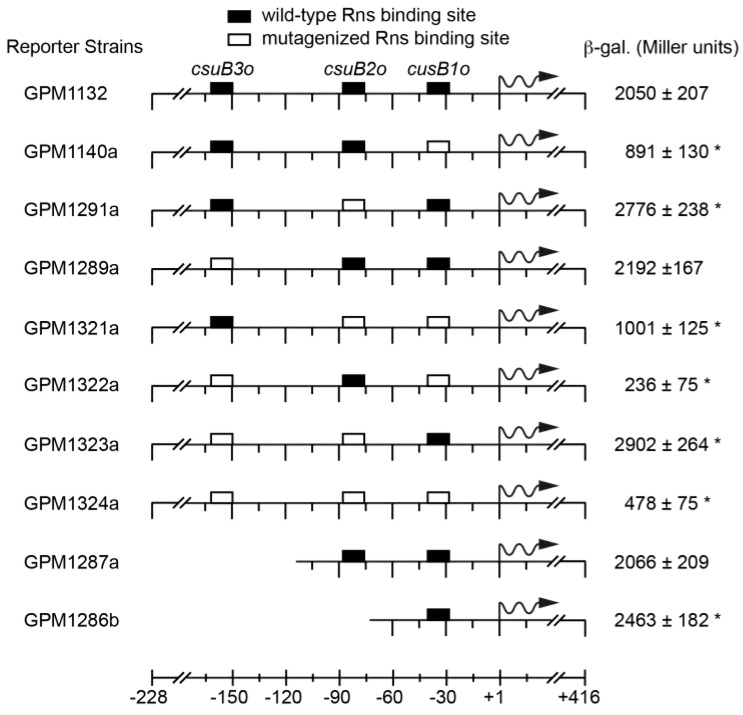
Analyses of Rns binding sites in vivo. Lac reporter strains were transformed with an Rns expression plasmid and the expression of β-galactosidase was quantified. Numbering is relative to the Rns-dependent transcription start site with wavy arrows indicating the direction of transcription. The average and standard deviation of Miller units are reported, *n* ≥ 3. * Statistically significant (*p* ≤ 0.005) when compared to GPM1132. In the absence of Rns, each reporter strain produced ≤20 Miller units.

In the absence of Rns, all reporter strains produced low levels of β-galactosidase expression (≤20 Miller units, data not shown). When strain GPM1132 was transformed with an Rns expression plasmid, the expression from the wild-type promoter increased to 2050 Miller units, a ca. 300-fold increase above the basal level of expression (Figure 4). Mutagenesis of the promoter proximal site *csuB1o* significantly (*p* < 0.001) reduced, but did not abolish, Rns-dependent transcription from *csuBp* (Figure 4, see GPM1140a). In contrast, the mutagenesis or deletion of promoter distal site *csuB3o* did not significantly affect the level of expression from *csuBp* relative to the wild-type promoter (Figure 4, compare GPM1289a and GPM1827a to GPM1132). 

Interestingly, Rns-dependent expression from *csuBp* increased 1.3-fold when *csuB2o* was mutagenized (Figure 4, see GPM1291a). A similar increase was observed when both *csuB2o* and *csuB3o* were mutagenized (Figure 4, see GPM1323a). Likewise, deletion of *csuB2o* also resulted in an increase of β-galactosidase expression (Figure 4, compare GPM1287a to GPM1286b). Although these increases were small relative to GPM1132, they were nonetheless statistically significant. Likewise, a small increase was observed when both *csuB1o* and *cusB2o* were mutagenized relative to GPM1140a harboring only mutagenized *cusB1o*. Although this latter result was not statistically significant, these results suggest that occupancy of *csuB2o* may have a slight inhibitory effect upon Rns-dependent expression from *csuBp*. At the very least, *csuB2o* appears to contribute relatively little towards the activation of *csuBp*. In contrast, mutagenesis of the promoter distal site significantly decreased the expression of β-galactosidase but this effect was only observed when *csuB1o* was also mutagenized (Figure 4, compare GPM1322a to GPM1289a). Mutagenesis of all three binding sites did not decrease expression further, and in fact strain GPM1324a had a small increase over GPM1322a. 

## 4. Discussion

In this study we have shown that the expression of the CS14 pilus is positively regulated by the ETEC virulence regulator Rns. Although three Rns binding sites were identified upstream of the CS14 pilus promoter, mutagenesis of the promoter proximal site had the greatest impact upon Rns-dependent transcription from *csuBp*. This is not surprising because the occupancy of site *csuB1o*, which is centered at −34.5, would place Rns in a position to make numerous productive contacts with RNA polymerase. Interestingly, the occupancy of site *csuB2o* centered at −80.5 seemed to correlate with a slight inhibition of Rns-dependent transcription which was relieved when *csuB2o* was mutagenized. This was unexpected because the mutagenesis of promoter distal sites at other Rns-dependent pilin promoters decreases Rns-dependent transcription [27,28]. However, in these latter cases the second Rns binding site is positioned ca. 30 bp further upstream than *csuB2o*. Thus, it is possible that the position of the second Rns binding site determines if occupancy of the site will have negative or positive consequences with regards to transcription. Based upon other well-characterized activators, positive consequences from promoter distal sites could result from interactions between Rns and the carboxy-terminal domain of the α subunit of RNA polymerase [42]. However, the mechanism of the negative effect is less clear and would require additional experimentation to elucidate. 

The presence of a third Rns binding site also differentiates the CS14 promoter from CS1, CS17, CS19, and PCFO71 pilus promoters which have only two Rns binding sites [27,28]. However, the CS14 distal site (*csuB3o*) appeared to be a low-affinity binding site and mutagenesis of it had no effect on promoter activity as long as the promoter proximal site (*csuB1o*) was intact. Only in the absence of *csuB1o* did *csuB3o* contribute towards activation of *csuBp*. The distance of *csuB3o* centered at −155.5 from *csuBp* is near the upper limit for activator interactions with the carboxy-terminal domain of the α subunit of RNA polymerase [43]. However, DNA bending or looping may decrease the distance considerably and facilitate productive interactions between Rns bound at *csuB3o* and RNA polymerase. Despite these remaining mechanistic questions, we have definitively shown that Rns activates the CS14 pilin promoter. CS14 pili belong to class 5 or the α-clade of ETEC pili. Thus, members of this clade are related by both pilin phylogeny and similar regulatory control, as CS1, CS2, CS4, CS17, CS19, and CFA/I pili are all positively regulated by Rns [22,25,27,36,40]. The CS5 pilus is likely to be the only exception within this clade because our sequence analyses suggest that the CS5 promoter lacks Rns binding sites.

## Figures and Tables

**Table 1:** Bacterial strains.

*E. coli* Strain	Relevant Genotype	Notes
MC4100	∆(*argF-lac*)U169	K-12; gift from J. Scott (Emory University, Atlanta, GA, USA)
WS3294A	*csuBA1A2CD*	ETEC CS14^+^ ST^+^, human clinical isolate; gift from S. Savarino (Naval Medical Research Center, Silver Spring, MD, USA)
GPM1124	*attB*_HK022_::pCS14Lac1	*csuBp* −228 to +117 ^1^, wild-type *
GPM1132	*attB*_HK022_::pCS14Lac2	*csuBp* −228 to +461, wild-type *
GPM1140a	*attB*_HK022_::pCS14Lac4	*csuBp* −228 to +461, mutagenized csuB1o *
GPM1291a	*attB*_HK022_::pCS14Lac3	*csuBp* −228 to +461, mutagenized cusB2o *
GPM1289a	*attB*_HK022_::pCS14Lac7	*csuBp* −228 to +461, mutagenized csuB3o *
GPM1321a	*attB*_HK022_::pCS14Lac8	*csuBp* −228 to +461, mutagenized *csuB1o*, csuB2o *
GPM1322a	*attB*_HK022_::pCS14Lac9	*csuBp* −228 to +461, mutagenized *csuB1o*, csuB3o *
GPM1323a	*attB*_HK022_::pCS14Lac10	*csuBp* −228 to +461, mutagenized *csuB2o*, csuB3o *
GPM1324a	*attB*_HK022_::pCS14Lac11	*csuBp* −228 to +461, mutagenized *csuB1o*, *csuB2o*, csuB3o *
GPM1287a	*attB*_HK022_::pCS14Lac6	*csuBp* −114 to +461, deleted csuB3o *
GPM1286b	*attB*_HK022_::pCS14Lac5	*csuBp* −73 to +461, deleted *csuB2o*, csuB3o *

^1^ Relative to Rns-dependent transcription state site. * From this study.

**Table 2:** Oligonucleotide primers.

Oligo. ID	Oligonucleotide Sequence ^1^
39	TTTGAATTCTTTAAATCTTTTGATA
542	GATGGATCCCCTGCATCAATCGATGAG
554	GCAGAATTCGGTAAAGATCTGATTAGAGCCGC
346	GGATATATCATAAAGTTTGCATTTG
566	TGACTAGTTTTTTTATCTCATTTTTTTTTG
567	AACTAGTCAATGCAATAAATCATCTGTG
564	GTTGGTACCAAAAATAGCACAGATGACACAG
565	TTGGTACCAATACTGCGATGTTCATTG
769	GTGAAACCGGTTCCTTTTTCAGGTTTTTTTAATC
770	GAGGAACCGGTTTCAACACTCTCTGTGCGC
771	GTAGGATCCCTATCAATGAACATC
772	GTAGGATCCACAGATGACACAGATG

^1^ All sequences are written 5′ to 3′ with primer/template mismatches underlined. Primers were synthesized by Sigma-Aldrich, St. Louis, MO, USA.

## Abbreviations

The following abbreviations are used in this manuscript:

MBP	maltose binding protein
CRP	cAMP receptor protein
FDA	United States Food and Drug Administration
